# Urethra contouring on computed tomography urethrogram versus magnetic resonance imaging for stereotactic body radiotherapy in prostate cancer

**DOI:** 10.1016/j.ctro.2023.100722

**Published:** 2023-12-30

**Authors:** Wee Loon Ong, M. Allan Hupman, Melanie Davidson, Mark Ruschin, Jay Detsky, Stanley Liu, Danny Vesprini, Andrew Loblaw

**Affiliations:** a Odette Cancer Centre, Sunnybrook Health Sciences Centre, Toronto, Canada; b Department of Radiation Oncology, University of Toronto, Canada; c Alfred Health Radiation Oncology, Monash University, Melbourne, Australia; d Department of Health Policy, Measurement and Evaluation, University of Toronto, Canada

**Keywords:** Prostate SBRT, Urethra contour, Urethrogram, MRI sequences

## Abstract

Accurate urethra contouring is critical in prostate SBRT. We compared urethra contouring on CT-urethrogram and T2-weighted MRI. The dice similarity coefficient, Jaccard index, Hausdorff distance and mean distance to agreement were evaluated. All four metrics indicate better agreement and less variability in urethra contouring on CT-urethrogram, compared to T2-weighted MRI.

## Highlights

• Urethra is increasingly recognized as an organ-at-risk for prostate SABR.
• Accurate urethra contouring is critical to reduce GU toxicities.
• We compared urethra contouring on CT-urethrogram and T2-weighted MRI.
• There is better agreement and less variability in urethra contouring on CT-urethrogram.

## Introduction

There is increasing recognition that the urethra is an important organ at risk in stereotactic body radiotherapy (SBRT) for prostate cancer [1], and that increased radiation dose to the urethra is associated with a higher risk of genitourinary toxicities [2]. This is particularly important if a simultaneous boost to the dominant intra-prostatic lesion (DIL) is given, as the urethra could be receiving escalated doses [3]. As such, several prostate SBRT protocols have called for urethra-sparing measures in prostate SBRT [4], [5], [6], [7], [8], with suggested urethra dose constraints for different prostate SBRT fractionations [9], [10].

However, this is contingent on accurate urethra contouring. Currently there is no consensus guideline for urethra contouring [11]. Visualisation of the prostatic urethra on computed tomography (CT) is poor, making delineation challenging and fraught with uncertainty. A Foley catheter can be inserted to help localize and visualize the urethra on CT [4]. However, this can deform the urethra and the catheter insertion needs to be repeated at each SBRT fraction, which can be invasive and introduce the risk of infection [12], [13]. An earlier study investigated the use of Nickel-Titanium stent that is left in situ in the prostate for the course of radiotherapy, which can be used to guide urethra contouring [14]; however, this is an invasive procedure and there is the risk of stent dislocation [15]. An alternative option is to perform a urethrogram at the time of planning CT [6], which relies on radiopaque contrast to enhance the appearance of the urethra on CT. This strategy has been used in our institutional prospective trials, including the 5STAR [16] and 2SMART [17] trials. Magnetic resonance imaging (MRI), which provides superior soft tissue contrast may improve accuracy in urethra contouring, as the urethra appears hyper-intense on T2-weighted MRI [11], [18]. In this study, we aim to evaluate the inter-observer variation and agreement in urethra contouring based on CT-urethrogram and T2-weighted MRI.

## Methods

### Study cohort

This is a retrospective study using data of ten patients enrolled in the 2SMART trial (NCT03588819), a phase 2 prospective trial of two-fraction prostate SBRT with focal boost to the MRI-defined DIL [17]. All patients in the 2SMART trial had a retrograde urethrogram done at the time of planning CT. The patient was positioned supine with the rectal immobilisation device (GU-Lok [19]) inserted. The penile tip was cleaned and approximately 2cc iodinated contrast with 10cc lidocaine jelly was slowly injected into the penile urethra. A penile clamp was applied to reduce the leakage of contrast. A planning CT scan with 2 mm slice thickness was performed. A separate multi-parametric MRI (mpMRI, which included T1 and T2-weighted images, diffusion-weighted images, and dynamic contrast enhancement) was obtained, without GU-Lok or urethrogram. The CT images were fused with mpMRI for target volume delineation.

### Urethra contouring

For each of the ten cases, five genitourinary radiation oncologists (‘observers’), with a median of 14 years in practice (range: 2–23 years), independently contoured the prostatic urethra in MIM 7.2.8 (MIM Software Inc) on the anonymized CT dataset with urethrogram and MRI datasets (including T2-weighted axial, sagittal, and coronal). As per the 2SMART trial protocol, the urethra was contoured with a minimum 6 mm pearl. For each case, a consensus contour was generated using the *Simultaneous Truth of Performance Level Estimation* (STAPLE) function within the Computational Environment for Radiotherapy Research (CERR) in MATLAB version 2019b (Mathworks, Natwick, MA) [20] to provide a probabilistic estimate of the ‘reference contour’ representing the ‘true’ urethra anatomy. The STAPLE (i.e., reference) urethra contour was generated for urethra contours done on CT-urethrogram and MRI for each case.

### Metrics evaluation

Each observers’ contours were compared to the STAPLE contours to assess for interobserver variability, separately for the CT-urethrogram dataset and MRI dataset. In addition, the STAPLE contours on CT-urethrogram vs MRI for each case were compared to assess the variation and/or agreement between the two different imaging modalities. Two overlap metrics, Dice Similarity Coefficient (DSC) and Jaccard Index (JI), and two distance metrics, the Hausdorff distance (HD) and mean distance to agreement (MDA), were computed for each comparison.

• ***Dice Similarity Coefficient (DSC)*** is a widely used metric to evaluate spatial overlap between multiple contours in radiation oncology settings, by comparing the intersection and the union of two contours [21]. The dice similarity coefficient value ranged from 0 (no overlap) to 1 (complete overlap). A dice similarity coefficient score of > 0.70 has been reported as demonstrating ‘good’ spatial and volumetric similarity [21].
• ***Jaccard index*** provides a measure of overlap between datasets by comparing the intersection of the two sets and their union (e.g., percent overlap), with value of 0 being completely separate and 1 being the same. The dice similarity coefficient emphasizes the intersection of contours, while the Jaccard index prioritizes contour differences.
• ***Hausdorff distance*** is a measure of the greatest distance from a point on one contour to the closest point on another contour and is an indication of how far the two contours are apart (or rather, the maximum discrepancy between contours). Higher value represents more variability in contour. Since Hausdorff distance gives the maximum distance between contours, it is sensitive to outliers.
• ***Mean distance to agreement*** is a measurement of the average overall distance between the contours, with lower value representing better agreement in contour. Of note, small mean distance to agreement values obtained from averaging random errors may not be distinguishable from small systematic differences in the overall volume and position.

## Results

**Fig. 1:**
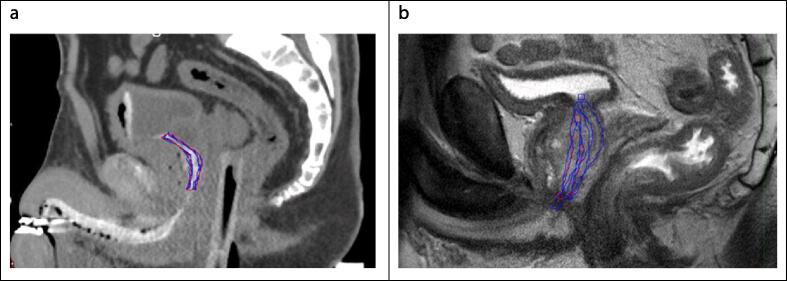
Sagittal example dataset with observer urethra contours (blue line) and reference contour generated based on STAPLE (red line) on CT urethrogram (a), and mpMRI (b). (For interpretation of the references to colour in this figure legend, the reader is referred to the web version of this article.)

Fig. 1 showed two examples of individual observers’ urethra contour (in blue) and the STAPLE contour (in red) on CT-urethrogram dataset (Fig. 1a) and MRI dataset (Fig. 1b).

When comparing the five observers’ urethra contours against the STAPLE contours for the ten cases, the mean and median dice similarity coefficient were 0.81 (SD = 0.03) and 0.82 (range = 0.76–0.86) in the CT-urethrogram dataset and 0.62 (SD = 0.06) and 0.61 (range = 0.52–0.73) in the MRI dataset (Table 1), indicating better agreement of the urethra contours using the CT-urethrogram dataset. The mean and median Jaccard index was 0.66 (SD = 0.04) and 0.67 (range = 0.58–0.70) in the CT-urethrogram dataset and 0.46 (SD = 0.06) and 0.45 (range = 0.38–0.45) on MRI dataset; again, indicating better agreement in urethra contouring using the CT-urethrogram dataset. The mean and median Hausdorff distance was 2.85 mm (SD = 0.60) and 2.75 mm (range = 2.08–4.21) in the CT-urethrogram dataset and 4.83 mm (SD = 0.67) and 4.71 mm (range = 3.76–5.69) in the MRI dataset, indicating that the observers’ urethra contour were further from the STAPLE contour in the MRI dataset. In supporting this, the mean and median distance to agreement was 0.49 mm (SD = 0.09) and 0.52 mm (range = 0.37–0.60) in the CT-urethrogram dataset, and 1.06 mm (SD = 0.27) and 1.05 mm (range = 0.70–1.57) in the MRI datasets.

**Table 1:** Metrics comparing individual observers’ urethra contour (n = 5) and STAPLE contour on CT-urethrogram and T2-weighted MRI.

	**Dice similarity coefficient**		**Jaccard index**		**Hausdorff distance (mm)**		**Mean distance to agreement (mm)**	
**Patient**	**CT-u**	**MRI**	**CT-u**	**MRI**	**CT-u**	**MRI**	**CT-u**	**MRI**
**1**	0.83	0.65	0.66	0.48	3.28	3.76	0.53	0.74
**2**	0.77	0.73	0.60	0.54	2.67	4.42	0.51	0.70
**3**	0.83	0.59	0.67	0.41	3.14	5.69	0.60	1.22
**4**	0.81	0.59	0.65	0.42	2.40	4.73	0.53	0.97
**5**	0.82	0.59	0.69	0.42	2.62	4.14	0.40	0.95
**6**	0.86	0.70	0.70	0.53	2.08	5.08	0.37	1.13
**7**	0.76	0.57	0.58	0.40	4.21	5.60	0.60	1.32
**8**	0.80	0.63	0.69	0.48	2.94	4.49	0.39	1.12
**9**	0.84	0.64	0.69	0.49	2.36	4.68	0.41	0.92
**10**	0.79	0.52	0.62	0.38	2.82	5.67	0.57	1.57
**Mean (SD)**	0.81 (0.03)	0.62 (0.06)	0.66 (0.04)	0.46 (0.06)	2.85 (0.60)	4.83 (0.67)	0.49 (0.09)	1.06 (0.27)
**Median (range)**	0.82 (0.76–0.86)	0.61 (0.52–0.73)	0.67 (0.58–0.70)	0.45 (0.38–0.54)	2.75 (2.08–4.21)	4.71 (3.76–5.69)	0.52 (0.37–0.60)	1.05 (0.70–1.57)

CT-u = computed tomography with urethrogram; MRI = magnetic resonance imaging; SD = standard deviation

In addition, when comparing the STAPLE contours between CT-urethrogram and MRI, the mean dice similarity coefficient was 0.46 (SD = 0.15), the mean Jaccard index was 0.30 (SD = 0.12), the mean Hausdorff distance was 5.4 mm (SD = 1.62) and the mean distance to agreement was 1.34 mm (SD = 0.50).

## Discussion

There is increasing recognition of the need to limit radiation dose to the urethra in prostate SBRT, and hence accurate and reproducible urethra contouring is critical. In this study, we compared the agreement and variability in urethra contouring based on CT-urethrogram and T2-weighted MRI. While there have been several studies evaluating urethra contouring on different imaging modalities and MRI sequences [18], [22], [23], to our knowledge, this is the first study that compared *CT-urethrogram* with MRI. Despite some degree of variability between the five observers in all ten cases in this contouring study, all evaluated metrics consistently point towards better agreement and less variation in urethra contouring based on CT-urethrogram, compared to standard T2-weighted MRI.

The findings of our study has important clinical implications. Currently, prostate SBRT planning process generally requires a planning CT scan, which is then fused with MRI to guide contouring of the prostate. Our findings showed that CT-urethrogram allows excellent urethra visualization with high agreement in urethra contouring (mean dice similarity coefficient of > 0.8). Earlier similar study by Richardson et al showed much lower agreement in urethra contouring using CT alone (without urethrogram), with mean dice similarity coefficient of 0.47 [22]. The use of urethrogram in prostate radiotherapy planning is not new. It has been used to identify the inferior border or apex of prostate in the days of field-based radiotherapy for prostate cancer and is generally well-tolerated [24]. While the urethrogram is less likely to ‘deform’ the urethra compared to the use Foley catheter [12], the extent to which the per-urethra contrast injection may impact on the intra-prostatic urethra position is unknown, and has never been quantified.

While T2-weighted MRI provides better soft tissue contrast and is commonly used to guide prostate contouring, our findings showed that the urethra is in fact better visualized, with less variability in contouring, on CT-urethrogram compared to T2-weighted MRI. Reassuringly, the inter-observer variability in urethra contouring on MRI in our study (mean dice similarity coefficient of 0.61) is not dissimilar to prior studies (mean dice similarity coefficient of 0.61) [22], suggesting our findings are not due to ‘poorer’ urethra contouring on MRI among the observers. However, as we move towards MRI only workflow [25] or treatment on MR-Linac (i.e., Linac with on-board MRI) [26], a planning CT may no longer be performed as part of prostate SBRT planning. In this situation, additional urethra-optimized MRI sequences (in addition to the standard T2-weighted MRI used for prostate contouring) will be required to better visualize the urethra. In the study by Richardson et al, the use of urethra-optimized T2-weighted Sampling Perfection with Application optimized Contrasts using different flip angle Evolution (T2-SPACE) improved agreement in urethra contouring with the dice similarity coefficient to 0.78 [22]. Other sequences such as the 3D half Fourier acquisition single-short turbo spine echo (3D-HASTE) [18], post-urination MRI sequence [23], and use of micturating urethrography [27] have also been investigated.

It is important to acknowledge that there is no consensus on best methodology for contouring comparison [28]. A major strength of our study is the use of combination of different metrics, including overlap and distance metrics, to assess the variation and agreement in urethra contouring. Clinically important differences in spatial position of contour boundaries may not be fully captured by indexes such as dice similarity coefficient and Jaccard index. This is especially the case with relatively small volume contours such as urethra. Also, a mis-identification in position in a portion of the urethra contour may be more meaningful compared to overall overlap agreement, particularly in the context of dose-painting in the vicinity of urethra.

There are several limitations in this study. The ‘reference’ contour was generated using the STAPLE methods, taking into account contours by all five observers, including outliers, and this may result in a biased ‘reference’ contour. In fact, when we compare the STAPLE contours generated on CT-urethrogram dataset vs MRI dataset, there is also high variability. However, STAPLE is a well-established and commonly used methodology to generate a ‘reference’ or ‘consensus’ contour in other contouring studies [29], [30]. A T2-weighted MRI sequence was used in this study, which may not necessarily be optimal for urethra visualisation. However, it is important to note that not every centres have the experiences or resources for dedicated urethra-optimised MRI sequences, and the MRI sequences used in this study is reflective of common MRI sequences acquired as part of diagnostic prostate MRI. Also, a relatively small number of cases (n = 10) were included in this study. The findings of our study is reflective of the contouring by the five observers (of varying experience) and may not be generalized to other observers. Nonetheless, the consistent findings across all ten patients compared using multiple metrics suggest that increasing the number cases or observers is unlikely to lead to a change in the findings.

## Conclusion

Our study showed that CT-urethrogram allows for better visualization of the urethra resulting in better agreement and less variability in urethra contouring compared to standard T2-weighted MRI used in prostate contouring. With increasing move towards urethral-sparing prostate SBRT, we believe that CT-urethrogram should be considered as part of planning CT in prostate SBRT to guide urethra delineation. At the same time, with the shift towards MRI-only radiotherapy planning workflow, additional MRI imaging protocols and sequences will be required to improve urethra visualisation on MRI for more accurate and consistent delineation of urethra.

## Funding

The study was funded by the Tolmar Canada & Sunnybrook Prostate Care Quality Improvement Initiative Grant.

## Data sharing statement

Research data is stored in institutional repository and will be shared upon request to the corresponding author.

## CRediT authorship contribution statement

**Wee Loon Ong:** Conceptualization, Data curation, Funding acquisition, Methodology, Writing – original draft, Writing – review & editing. **M. Allan Hupman:** Conceptualization, Data curation, Formal analysis, Funding acquisition, Methodology, Software, Writing – review & editing. **Melanie Davidson:** Conceptualization, Funding acquisition, Methodology, Software, Writing – review & editing. **Mark Ruschin:** Methodology, Software, Writing – review & editing. **Jay Detsky:** Conceptualization, Writing – review & editing. **Stanley Liu:** Conceptualization, Writing – review & editing. **Danny Vesprini:** Conceptualization, Writing – review & editing. **Andrew Loblaw:** Conceptualization, Funding acquisition, Methodology, Supervision, Writing – review & editing.

## Declaration of competing interest

The authors declare that they have no known competing financial interests or personal relationships that could have appeared to influence the work reported in this paper.
